# Research progress of periostin and osteoporosis

**DOI:** 10.3389/fendo.2024.1356297

**Published:** 2024-02-29

**Authors:** Chuyue Yuan, Junyan Li

**Affiliations:** Department of Endocrinology and Metabolism, Heji Hospital Affiliated to Changzhi Medical College, Changzhi, China

**Keywords:** periostin, osteoporosis, bone metabolism, bone formation, cortical bone

## Abstract

Periostin, as a unique extracellular matrix, is mainly produced during ontogeny and in adult connective tissues that bear mechanical loads, such as heart valves, skin, periodontal ligaments, tendons, and bones. By binding to the integrin on the cell surface and activating Wnt/β-catenin, NF-κB, Fak and other signaling pathways, it regulates the tissues *in vivo* positively or negatively, and also has different effects on the occurrence and development of various diseases. Periostin is an important factor, which can promote cell proliferation, stimulate tissue repair and maintain the integrity of the structure and function of connective tissue. It also promotes the formation, regeneration and repairation of bone. Recent studies have shown that periostin is important in bone metabolic diseases. The increased expression of periostin can affect bone mineral density at different sites, and its relationship with traditional biochemical markers of bone turnover has not been conclusively established. This article reviews the research results and potential applications of periostin in osteoporosis.

## 1 Introduction

Osteoporosis (OP) is a systemic bone disease characterized by low bone mass and destruction of bone microstructure, leading to increased bone fragility and fracture (1). It is a complex disease with interactions between genetic and environmental factors. At present, the gold standard for the diagnosis of osteoporosis is the dual-energy X-ray absorptiometry (DXA) scanning (2, 3). Although DXA is of great value in clinical diagnosis, but the evaluation of bone mineral density can only distinguish the population into healthy population, osteopenia population and osteoporosis population, cannot distinguish the causes of osteoporosis (4). Bone turnover markers (BTMs) cannot be used to diagnose osteoporosis, but they can reflect bone remodeling earlier, which plays an important role in the diagnosis and differential diagnosis of bone metabolic diseases, the prediction of fracture risk and the evaluation of efficacy evaluation (5). However, BTMs which commonly used in clinic lack specificity for bone tissue, they cannot distinguish the bone turnover between cortical and cancellous bone, cannot reflect the activity of osteocytes, and is difficult to reflect bone quality and evaluate bone fragility (6).

Periostin is generally considered as a potential marker of several skeletal and non-skeletal diseases, including lung, asthma, allergy, liver, diabetes, renal function, or cancer (7–14). In view of the possible diagnostic and therapeutic value of periostin in a variety of diseases, periostin may become a new clinical biochemical marker of bone turnover and play an important role in exploring the pathogenesis, treatment methods and potential therapeutic targets of osteoporosis. Therefore, this review summarizes the roles of periostin in the regulation of osteogenic differentiation, bone mineralization, bone mechanical response, bone repair, and the activity and apoptosis of bone-related cells to determine the research achievements, potential applications and challenges of periostin in osteoporosis.

## 2 Periostin

**Figure 1:**
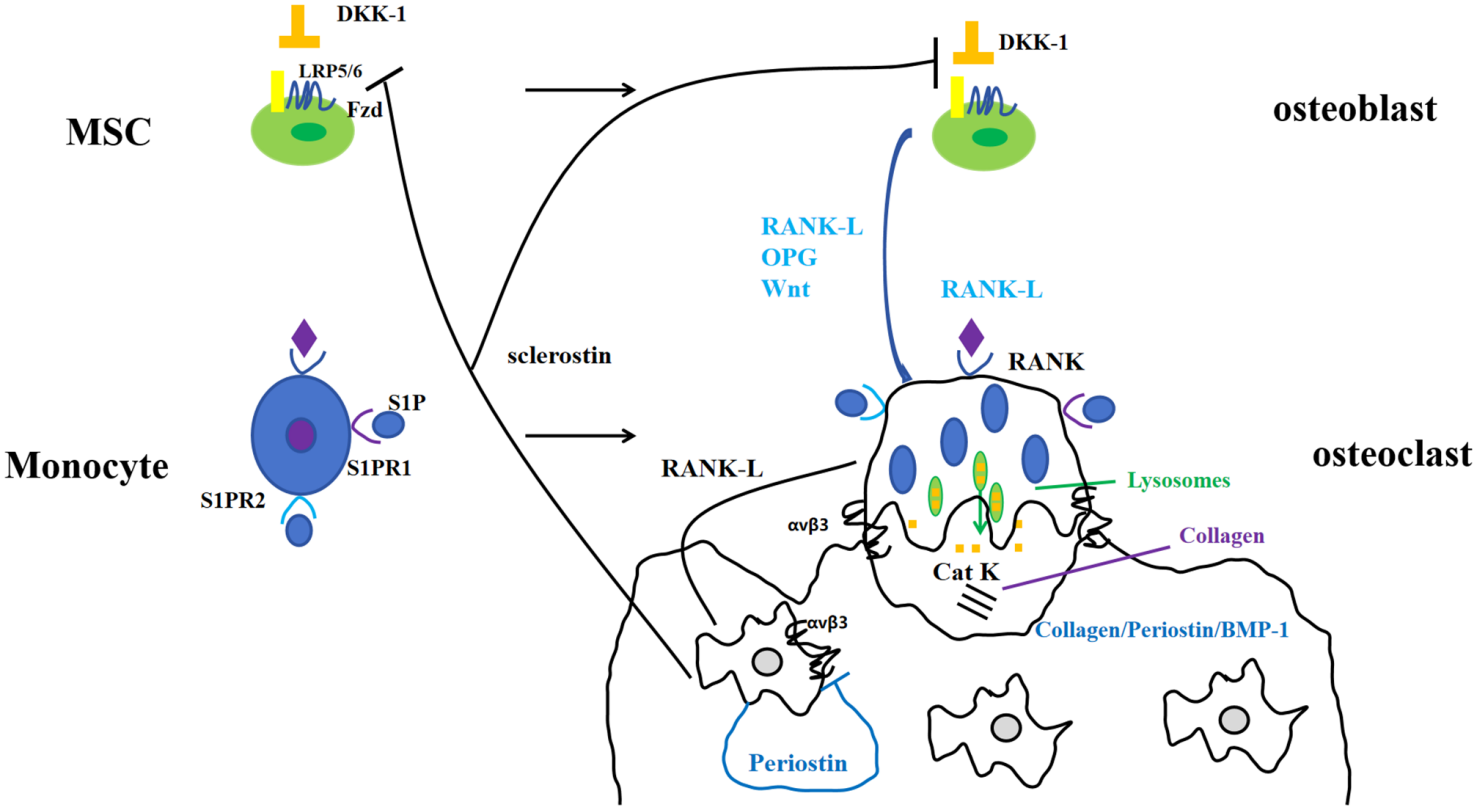
Periostin and bone metabolism-signaling pathways.

**Figure 2:**
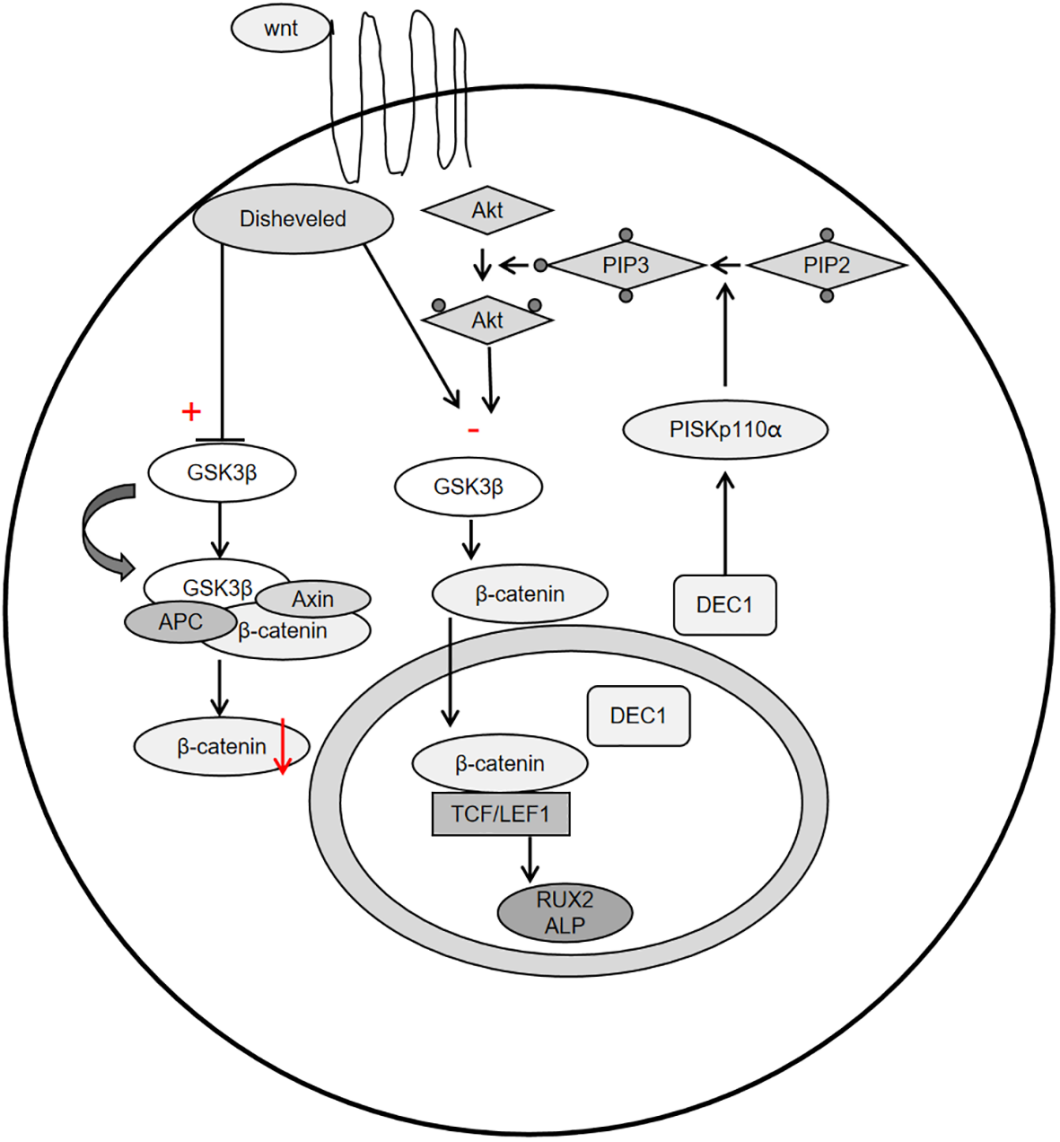
Schemata of the classical Wnt signaling pathway, PI3K-Akt-GSK3β-β-catenin signaling pathway.

Periostin, also known as osteoblast-specific factor 2 (OSF-2), is a bone adhesion molecule cloned from the cDNA library of osteoblast cell line MC3T3-E1 by Takeshita et al. It has a molecular weight of 90-kD (15). As a unique extracellular matrix protein, is mainly produced during ontogeny, as well as in adult connective tissues subjected to mechanical loads, such as heart valves, skin, periodontal ligaments, tendons, and bones (7). Periostin contains vitamin K-dependent γ-carboxyglutamic acid residues at one end, it is a kind of Vitamin K-dependent proteins (16). It binds to integrin receptors αvβ3 and αvβ5 on the cell surface to trigger Wnt/β-catenin, NF-κB/STAT3, PI3K/Akt and focal adhesion kinase signaling pathways and regulates the expression of downstream genes (**Figures 1**, **2**). It plays an important role in promoting osteoblast differentiation and survival, cell adhesion, tissue repair and in maintaining the integrity of connective tissue structure and function (17–19).

### 2.1 Role of periostin in bone metabolism

#### 2.1.1 Periostin affects osteogenesis and promotes bone regeneration

Merle et al. (20) studied the expression of periostin mRNA in mouse calvarial osteoblast-like cell line MC3T3-E1 and *in vitro* differentiated mouse long bone osteoclasts, and detected the secretion of periostin by ELISA, which confirmed that periostin appeared at the early stage of osteoblast differentiation. If periostin was blocked, Runx2/Cbfa1 was significantly down-regulated, and finally affected the differentiation of osteoblasts. Therefore, Merle et al. suggested that periostin could be considered as a potential biomarker for early osteoblast differentiation and new bone formation. Kudo et al. (21) found that bone formation was increased in mice which overexpress periostin, whereas mice deficient in periostin showed stunted growth and low cortical and reticular bone, as indicated by reduced bone mineral density. Li et al. (22) isolated bone marrow mesenchymal stem cells (BMMSCs) from ovariectomized rats and normal rats. Through the expression of periostin and osteogenesis in ovariectomized rats, it was found that the level of periostin in bone marrow mesenchymal stem cells isolated from ovariectomized rats was significantly decreased. *Postn* gene modified bone marrow mesenchymal stem cells can promote alveolar bone regeneration in ovariectomized rats, further demonstrating the correlation between periostin and bone formation. Another study on rats also found that in 6-week-old rats with periostin overexpression, the activity of osteoblasts was enhanced, the femoral bone formation and bone mass were significantly increased (23). The role of periostin in regulating periosteal bone was also age-related. The formation of periosteal bone was significantly reduced in young and adult *Postn* ^-/-^ mice, but increased in old *Postn* ^-/-^ mice. Vitronectin can compensate for the reduced mineralization caused by periostin deficiency (24). In addition, *Postn* gene has also been shown to promote bone regeneration and calcification during mandibular distraction osteogenesis in rabbits (25). The expression of Runx2, RANKL and OPG in MC3T3-E1 cells after silencing periostin gene was detected, which further confirmed that the expression of Runx2 was reduced by silencing periostin gene, suggesting that periostin and Runx2 synergistically affect osteoblast differentiation (26).

#### 2.1.2 Periostin promotes collagen fiber formation, affects bone mineralization, and enhances bone strength

Some studies have found that after bone loss, the decrease of collagen fibers may be related to the decrease of periostin expression. The body can compensate by increasing the content of serum periostin, but the increased content is not enough to offset the loss of bone (27, 28). Several studies have shown that periostin can activate lysine oxidase, which is significantly down-regulated in calvarial osteoblasts of periostin-deficient mice, thereby affecting microfibrogenesis (29, 30). Periostin can also bind to mucin C, increase fiber branching, promote the formation of bone matrix network structure, and enhance bone strength. Periostin is more likely to bind to hydroxyapatite crystals, thereby promoting bone mineralization (16). However, there is a study found that periostin mRNA expression is negatively correlated with cell mineralization (31). Therefore, periostin can promote collagen fiber formation, but its effect on bone mineralization needs further study *in vitro* and *in vivo*.

#### 2.1.3 Periostin participates in the mechanical response of bone

Periostin is sensitive to the changes of mechanical stress, and the expression of periostin is increased under mechanical stress, overstimulation and injury. Experimental studies in animals have shown that reducing mechanical load can lead to damage of bone structure (trabecular bone and cortical bone), and this damage is accompanied by a reduction in periostin gene expression (32, 33). On the contrary, if the mechanical load is increased, such as increasing the axial compression load of the tibia of mice (25, 34) or high-intensity training (29), the secretion of periostin will be increased and sclerostin will be decreased (28, 30). In the limb of mice with relatively large activity, the expression of periostin increases, and the external diameter of bone and the mechanical properties of bone are also enhanced (35). Mechanical loading resulted in the overexpression of periostin (28, 30, 34–36). At present, there are few studies on reducing mechanical load in humans. The most recent population study on bone mass in astronauts found that although changes in bone mass and bone microstructure were observed after 4-6 months of spaceflight, the level of periostin did not change (37).

#### 2.1.4 Periostin promotes bone repair and affects callus formation

Periostin is important in all stages of bone repair, such as the initial activation of stem cells in the periosteum at the early stage of repair, the active phase of cartilage and bone deposition in fracture callus, and the final stage of bone bridge and stem cell bank reconstruction in the periosteum (38). Nakazawa et al. (39) used cDNA microarray method to find that periostin increased at fracture sites of mice with fractures compared with those without fractures, and reached the peak at 7 days after fracture and then decreased. In addition, the gene knockout mouse model showed that the microfracture density of *Postn* ^-/-^ mice was significantly higher than that before fatigue load, while *Postn* ^+/+^ mice had complete repair of microfracture on the 30th day after fatigue load, indicating that the expression level of periostin would affect the repair of fatigue fracture (40). It has also been proved that periostin can promote the proliferation and survival of human adipose tissue-derived mesenchymal stem cells, and stimulate angiogenesis by interacting with human adipose tissue-derived mesenchymal stem cells to synergistically promote the repair of skull defects (41). It has also been demonstrated that periostin expressing cells play a key role in intramedullary bone regeneration in a model of bone regeneration by surgical disruption (42). The important role of periostin in fracture healing is also age-dependent, with *Postn* expression reduced in fracture callus of aged mice compared with young mice (43).

These studies confirm that periostin, acting together with other factors, plays different roles in various processes of bone repair. Fracture healing and bone repair can increase periostin levels (34, 38, 41, 44) and up-regulate periostin mRNA (25), which can be observed up to a year after fracture (30), mainly after osteoporotic nonvertebral fractures (45).

In conclusion, periostin is a key factor in the regulation of bone microstructure. It can regulate the process of bone formation, promote the formation of collagen fibers, promote bone mineralization, enhance bone density, promote bone repair, and play an important role in the process of bone metabolism. However, it can better reflect the changes of cortical bone (46), and is also a risk factor for non-pyramidal fractures.

## 3 Periostin and osteoporosis

### 3.1 Periostin and traditional evaluation indicators of OP

Circulating periostin levels are stable from age 32 to 70 in healthy people (47), and serum periostin levels examined range from 36.1 to 133.3 ng/mL (48). As the substrate of cathepsin K, cathepsin K dependency periosteum protein fragment (K-*Postn*) level has been shown to predict low traumatic fractures. It is a predictor independent of bone mineral density, fracture risk assessment tool (FRAX), and bone turnover markers such as P1NP and CTX (49). There was experimental evidence proved that periostin is negatively correlated with bone mineral density (44, 50, 51), but there was although some confirmed the relationship between total periostin and bone mineral density was not significant (49, 52, 53). Yigitdol et al. found a negative correlation between periostin and lumbar spine DXA T-score in patients with primary hyperthyroidism (50). In postmenopausal women, serum periostin is negatively correlated with bone mineral density of lumbar spine (50), total hip (50) and femur (45). Kim et al. found that the serum periostin level in postmenopausal women was a risk factor for non-centrum fracture by studying the relationship between different fracture sites and serum periostin (45). Similar observation was made in patients with primary hyperparathyroidism, where bone isomers were measured, confirming that serum K-*Postn* was significantly associated with fractures in patients with primary hyperparathyroidism, but not with bone mineral density (52). It has also been confirmed that there is no significant difference in serum periostin levels in postmenopausal women with different bone mass, and there is no correlation between serum periostin levels and bone mineral density in different parts of the body (53) (**Table 1**).

**Table 1:** Previous studies on the correlation between periostin and bone mineral density.

Author,year,country	Study design	Number of patients	Age (years) (mean ± SD)/median (interquartile ranges)	Female(%)	BMD (g/cm^2^) Lumbar spine Total hip Femur (mean ± SD)/ median (interquartile range)	DXA T-score Lumbar spine Total hip Femur (mean ± SD)	Correlation between DXA T-score and periostin
Yan et al. (44), 2017, China	Case–control study	261	80 (76,84)	100	0.835(0.711,1.077) ND 0.689(0.576,0.925)	ND	Negative
Bonnet et al. (49), 2017, Switzerland	Case–control study	695	65.0 ± 1.5	100	0.97 ± 0.18 0.82 ± 0.12 0.68 ± 0.11	ND	NC
Yigitdol et al. (50), 2023, Turkey	Case–control study	40	50.4 ± 14.0	87.5	ND	-2.5 ± 0.26 ND ND	Negative
Guo et al. (51), 2022, China	Case–control study	385	65.7 ± 9.7	100	0.86(0.79–0.94) 0.75 ± 0.12 0.71 ± 0.11	ND	Negative
Pepe et al. (52), 2021, Italy	Case–control study	25	68.64 ± 5.98	100	0.818 ± 0.122 0.715 ± 0.08 0.613 ± 0.108	-2.07 ± 1.14 -1.9 ± 0.68 -2.17 ± 0.83	NC
Li et al. (53), 2017, China	Case–control study	261	61.3 ± 4.2	100	0.749 ± 0.079 0.774 ± 0.097 0.625 ± 0.078	ND	NC

ND, not determined; NC, not correlation.

Changes in serum periostin were not associated with changes in traditional BTMs, such as osteocalcin, bone specific alkaline phosphatase, type I procollagenamino-terminal peptide, and type I collagen carboxy-terminal peptide (49, 53). However, the relationship between periostin and bone mineral density and serum BTMs is still controversial.

### 3.2 OP treatment and periostin changes

Research on how osteoporosis treatment alters serum periostin levels is inconclusive, with only research prove zoledronic treatment has no effect on serum periostin levels (54). And there are a few papers showing that teriparatide treatment is able to increase periostin secretion, but it is not known whether this increase mediates the drug’s effect on bone, but it is not clear whether periostin increase associated with drug to increase bone mass (55).

## 4 Summary and prospect

In summary, periostin, as an extracellular matrix protein, exerts different effects by activating different pathways through binding to cell surface protein receptors. It can be expressed in a variety of tissues and cells, and plays an important role in bone formation, regeneration, repair, etc. It can promote bone mineralization, bone repair, and is particularly important in bone metabolism. Although there are significant progresses have been found, but the relationship between periostin and osteoporosis remains unclear. Therefore, as a new generation of biomarkers and possible therapeutic targets, its mechanism in osteoporosis needs to be further studied. It is hoped that in the near future, new research on periostin and osteoporosis will lead to useful diagnostic value and effective treatment to improve people’s quality of life.

## Author contributions

CY: Writing – review & editing. JL: Supervision, Writing – review & editing.
